# Supplementary material for: HIV incidence among sexually active young males and females in Kisumu County, Western Kenya
Source: PLoS One. 2026 May 22;21(5):e0349111. doi: 10.1371/journal.pone.0349111 (PMC13196977; doi:10.1371/journal.pone.0349111)
Supplement: Attachment — Submitted filename: Response to Reviewers_RV393 HIV Incid_Resub1 (clean)_signed.pdf [file pone.0349111.s001.pdf]

April 15, 2026

RE: HIV Incidence Among Sexually Active Young Males and Females in Kisumu County, Western Kenya (PLOS ONE PONE-D-25-59538)

Dear Editor,

Please find submitted with this cover letter our revised manuscript titled. We appreciate the constructive feedback from the Reviewers as well as the opportunity to revise and resubmit this manuscript.

On the pages that follow, we have listed our responses point-by-point to the comments and recommendations made by the reviewers. Line numbers refer to the clean copy of the revised manuscript. We would like to note that this work was supported by the Division of AIDS (DAIDS) at the U.S. National Institute of Allergy and Infectious Diseases and the Walter Reed Army Institute of Research. Through their representatives, the funders were involved in study design, study operation, data analysis, data interpretation, and writing of the report.

On behalf of all the authors of this manuscript, thank you for your consideration.

Sincerely,

Trevor A. Crowell, MD, PhD
Senior Director, Global Infectious Diseases | HJF
In support of MHRP, VDP, and DCB at the Walter Reed Army Institute of Research
Professor of Medicine, Division of Infectious Diseases, Uniformed Services University
6720A Rockledge Drive, Suite 400
Bethesda, MD 20817
Office: +1 301-500-3990
Cell: +1 301-275-6047

RESPONSE TO EDITOR AND REVIEWER COMMENTS

Journal Requirements:

When submitting your revision, we need you to address these additional requirements.
1. Please ensure that your manuscript meets PLOS ONE's style requirements, including those for file naming. The PLOS ONE style templates can be found at
https://journals.plos.org/plosone/s/file?id=wjVg/PLOSOne_formatting_sample_main_body.pdf
and
https://journals.plos.org/plosone/s/file?id=ba62/PLOSOne_formatting_sample_title_authors_affiliations.pdf
RESPONSE: Thank you, the style requirements were reviewed and the manuscript was adjusted accordingly.

2. Please include a complete copy of PLOS' questionnaire on inclusivity in global research in your revised manuscript. Our policy for research in this area aims to improve transparency in the reporting of research performed outside of researchers' own country or community. The policy applies to researchers who have travelled to a different country to conduct research, research with Indigenous populations or their lands, and research on cultural artefacts. The questionnaire can also be requested at the journal's discretion for any other submissions, even if these conditions are not met. Please find more information on the policy and a link to download a blank copy of the questionnaire here: https://journals.plos.org/plosone/s/best-practices-in-research-reporting. Please upload a completed version of your questionnaire as Supporting Information when you resubmit your manuscript.
RESPONSE: Thank you. The questionnaire has been completed and will be uploaded.

3. One of the noted authors is a group or consortium [RV393 Study Group]. In addition to naming the author group, please list the individual authors and affiliations within this group in the acknowledgments section of your manuscript. Please also indicate clearly a lead author for this group along with a contact email address.
RESPONSE: Thank you. The Acknowledgements have been updated as requested.

4. We note that you have indicated that there are restrictions to data sharing for this study. For studies involving human research participant data or other sensitive data, we encourage authors to share de-identified or anonymized data. However, when data cannot be publicly shared for ethical reasons, we allow authors to make their data sets available upon request. For information on unacceptable data access restrictions, please see http://journals.plos.org/plosone/s/data-availability#loc-unacceptable-data-access-restrictions.

Before we proceed with your manuscript, please address the following prompts:

a) If there are ethical or legal restrictions on sharing a de-identified data set, please explain them in detail (e.g., data contain potentially identifying or sensitive patient information, data are owned by a third-party organization, etc.) and who has imposed them (e.g., a Research

Ethics Committee or Institutional Review Board, etc.). Please also provide contact information for a data access committee, ethics committee, or other institutional body to which data requests may be sent.

b) If there are no restrictions, please upload the minimal anonymized data set necessary to replicate your study findings to a stable, public repository and provide us with the relevant URLs, DOIs, or accession numbers. Please see http://www.bmj.com/content/340/bmj.c181.long for guidelines on how to de-identify and prepare clinical data for publication. For a list of recommended repositories, please see https://journals.plos.org/plosone/s/recommended-repositories. You also have the option of uploading the data as Supporting Information files, but we would recommend depositing data directly to a data repository if possible.

Please update your Data Availability statement in the submission form accordingly.
RESPONSE: Thank you – We have no restrictions to sharing a de-identified data set. Please data set can now be accessed at https://dataverse.harvard.edu/dataset.xhtml?persistentId=doi:10.7910/DVN/1KECKH. The Data Availability statement will also be revised accordingly.

5. In the online submission form, you indicated that to request a minimal data set, please contact the Data Coordinating and Analysis Center (DCAC) at PubRequest@hivresearch.org and indicate the RV393 study along with the name of the manuscript.

All PLOS journals now require all data underlying the findings described in their manuscript to be freely available to other researchers, either 1. In a public repository, 2. Within the manuscript itself, or 3. Uploaded as supplementary information.
This policy applies to all data except where public deposition would breach compliance with the protocol approved by your research ethics board. If your data cannot be made publicly available for ethical or legal reasons (e.g., public availability would compromise patient privacy), please explain your reasons on resubmission and your exemption request will be escalated for approval.
RESPONSE: As noted in the response above, the de-identified data set has been uploaded to a public repository.

6. Thank you for stating the following financial disclosure:
This work was supported by a cooperative agreement (W81XWH-11-2-0174; W81XWH-18-2-0040) between the Henry M. Jackson Foundation for the Advancement of Military Medicine, Inc., and the U.S. Department of Defense. This research was funded, in part, by the U.S. National Institute of Allergy and Infectious Diseases.

Please state what role the funders took in the study. If the funders had no role, please state: "The funders had no role in study design, data collection and analysis, decision to publish, or preparation of the manuscript."
If this statement is not correct you must amend it as needed.

Please include this amended Role of Funder statement in your cover letter; we will change the online submission form on your behalf.
RESPONSE: Thank you – We confirm that the funders had no role in study design, data collection and analysis, decision to publish, or preparation of the manuscript.

7. Please amend your list of authors on the manuscript to ensure that each author is linked to an affiliation. Authors' affiliations should reflect the institution where the work was done (if authors moved subsequently, you can also list the new affiliation stating "current affiliation:…." as necessary).
RESPONSE: Thank you – We confirm that an affiliation is linked to each author.

8. One of the noted authors is a group or consortium [RV393 Study Group]. In addition to naming the author group, please list the individual authors and affiliations within this group in the acknowledgments section of your manuscript. Please also indicate clearly a lead author for this group along with a contact email address.
RESPONSE: Thank you – The Acknowledgements have been updated as requested.

9. If the reviewer comments include a recommendation to cite specific previously published works, please review and evaluate these publications to determine whether they are relevant and should be cited. There is no requirement to cite these works unless the editor has indicated otherwise.
RESPONSE: Thank you – The Reviewers made no such request.

10. Please review your reference list to ensure that it is complete and correct. If you have cited papers that have been retracted, please include the rationale for doing so in the manuscript text, or remove these references and replace them with relevant current references. Any changes to the reference list should be mentioned in the rebuttal letter that accompanies your revised manuscript. If you need to cite a retracted article, indicate the article's retracted status in the References list and also include a citation and full reference for the retraction notice.
RESPONSE: Thank you, we have reviewed and updated the references to ensure they are current and appropriate. To our knowledge, no cited article has been retracted.

Reviewers' comments:

Reviewer's Responses to Questions
Comments to the Author

1. Is the manuscript technically sound, and do the data support the conclusions?

The manuscript must describe a technically sound piece of scientific research with data that supports the conclusions. Experiments must have been conducted rigorously, with appropriate controls, replication, and sample sizes. The conclusions must be drawn appropriately based on the data presented.

Reviewer #1: Yes
Reviewer #2: Yes

---

2. Has the statistical analysis been performed appropriately and rigorously?
Reviewer #1: Yes
Reviewer #2: Yes

---

3. Have the authors made all data underlying the findings in their manuscript fully available?

The PLOS Data policy requires authors to make all data underlying the findings described in their manuscript fully available without restriction, with rare exception (please refer to the Data Availability Statement in the manuscript PDF file). The data should be provided as part of the manuscript or its supporting information, or deposited to a public repository. For example, in addition to summary statistics, the data points behind means, medians and variance measures should be available. If there are restrictions on publicly sharing data—e.g. participant privacy or use of data from a third party—those must be specified.
Reviewer #1: Yes
Reviewer #2: No

---

4. Is the manuscript presented in an intelligible fashion and written in standard English?

PLOS ONE does not copyedit accepted manuscripts, so the language in submitted articles must be clear, correct, and unambiguous. Any typographical or grammatical errors should be corrected at revision, so please note any specific errors here.
Reviewer #1: Yes
Reviewer #2: Yes

---

5. Review Comments to the Author

Please use the space provided to explain your answers to the questions above. You may also include additional comments for the author, including concerns about dual publication, research ethics, or publication ethics. (Please upload your review as an attachment if it exceeds 20,000 characters)

Reviewer #1:
1. Is the manuscript technically sound, and do the data support the conclusions?

Yes, this manuscript was technically sound and supported the conclusions. Overall, well written and put together and I did not identify any large issues. A few comments/questions regarding this section, specifically for the Methods section

A) On Page 6 it is listed that exclusion criteria include “significant conditions (medial, psychological/psychiatric, or social) that would interfere with study conduct”. There are then N=24 in Figure 1 who “Did not meet other eligibility requirements”. Are these the same group of

individuals? I would recommend clarifying Figure 1 to address this, potentially just with different labeling in the text box. Also, I would suggest that in the methods section that the authors try to clarify what that above statement actually means. Is Medial supposed to be “Medical”? Are there specific, objective Medical or Psychiatric conditions that the authors pre-identified that they thought would interfere with the study conduct or was each individual excluded based on best judgment by a single person or by a group of investigators? I think this part was little vague and could be written more clearly in order to best follow the methods section.
RESPONSE: Thank you for this helpful comment. We have corrected the typographical error. Upon review, 13 participants were excluded for “other eligibility requirements,” rather than 24 as originally presented. These included participation in another HIV prevention study (n = 1), anticipated unavailability for the 24-month follow-up period (n = 4), missing primary contact information (n = 1), missing alternative contact information (n = 2), and pre-existing medical or psychosocial conditions that could interfere with participation (n = 5). These categories were not mutually exclusive. We have updated the Figure 1 and have added a footer to outline the breakdown for those who “Did not meet other eligibility requirements”. Eligibility assessments were performed by a principal or sub-investigator, and exclusions were based on clinical judgment, rather than a predefined list of conditions. We have revised the Methods section to clarify this process by adding the following sentence:
***Determinations regarding significant underlying conditions were made by a principal or sub-investigator based on clinical judgment following review of each participant’s medical and psychosocial history, rather than a predefined list of conditions* (Lines 93-96).**

B) During the methods section and Figure 1, I was a little confused if the authors were following more of a Per-Protocol or Intention to Treat methodology for the Person-Time calculations as mentions in the statistics section on page 8 and 9 of the draft manuscript. As an example, Figure 1 lists three categories for individuals removed in follow up (decision to withdraw, death, lost to follow up). Were these individuals who were excluded included at all in the analysis if they completed questionnaires and HIV but withdrew, died, or were lost to follow up at the very end of the study (or over the COVID-19 pause mentioned in the paper)? It might be helpful to list just an additional sentence or two clarifying if any of these were used in analysis and if not, why was that decision made (could put later in the manuscript). I was also a little confused about what would happen to participants who initially did not have exclusion criteria but potentially gained exclusion criteria later in the study. As an example, if an individual was not pregnant at screening but after a time period became pregnant, how much of that was used in the analysis for Person-Time calculation? Once again, the method section was very strong and technically sound, but a few areas of clarity might help the manuscript read a little better.
RESPONSE: Thank you for this comment. We used an ITT-style person-time analysis in which all participants without HIV who completed at least one follow-up HIV test after enrollment contributed person-time until their last negative HIV test, after which they were censored. Participants who later withdrew, were lost to follow-up, or died were included in the analysis for the time they were observed. Participants who developed new conditions during follow-up (including pregnancy) were not removed and continued contributing person-time. The COVID-19 pause did not affect incidence estimates because no seroconversions occurred during that period. We clarified our approach in the Methods section as follows:

***All enrolled participants without HIV who completed at least one follow-up HIV test contributed person-time until their last documented negative test, after which they were censored* (Lines 135-137).**

2. Has the statistical analysis been performed appropriately and rigorously?
Yes, this manuscript has appropriate and rigorous statistical analysis. I only had a few comments on the statistical analysis section

A) It is very clear that authors only gathered behavioral survey data every 6 months (or up to 10 days after the HIV diagnosis) but I did not totally understand why the authors chose every 6 months versus every 3 months with the HIV testing? Was this just a logistical issue because of the burden of the survey? There is nothing wrong with this, but some clarification or justification might be beneficial. I think this is also important because the authors mention "recall and social desirability bias" on page 18, and I feel like adding length between the surveys likely added to this bias. Also, if a subject does test positive for HIV and then submits a survey, there might be a higher likelihood of recall bias if the individual already knows they have been recently diagnosed with HIV.
RESPONSE: Thank you for this comment. Behavioral questionnaires were administered every 6 months rather than every 3 months primarily due to the need to minimize participant burden within this high-mobility, high-risk population. We agree that a 6-month interval may increase recall and social desirability bias, as noted in the manuscript, and we have added this clarification to the Discussion as follows: *The 6-month interval for behavioral data collection, determined by participant-burden considerations, may have further contributed to recall and social desirability bias* (Lines 286-287).

B) On Page 9 of the manuscript, it states "Variables that were significant in the unadjusted bivariable model were included in a fully-adjusted multivariable model except for participant sex". I would recommend clarifying that the multivariable model adjusts for all the possible categorical variables. I think this is what was done in the statistical analysis, but it was not explicitly clear and could cause some confusion. Another option might be to edit Table 3 so the "Adjusted Relative Risk" column is a little more clear in the title or add this to the subtext you have on Page 15.
RESPONSE: We appreciate this comment as it helped us to identify a cause for confusion. In fact, the multivariable model did not adjust for all the possible categorical models. Instead, it adjusted for those variables that were statistically significant in the univariable model, and sex was forced into the model, regardless of statistical significance. The above quote was changed by deleting "fully" as follows: "… were included in an adjusted multivariable model except for participant sex". Additionally, in the Results section discussing the multivariable model, a sentence has been added that explicitly lists the variables selected: "Three variables were included in the multivariable Cox regression analysis: sex, number of sexual partners in the last 3 months, and forced to engage in unwanted sexual events". Lastly, on Table 3, "Relative Risk" was changed to "Hazard Ratio" as this was a typo.

3. Have the authors made all data underlying the findings in their manuscript fully available?
Yes, I believe all the data is fully available. I had no issues with the section. As mentioned above, it might be beneficial for the authors to clarify a few things in Figure 1 and data analysis in order to improve clarity for any readers; however, I feel like all the findings and data were clearly available.
RESPONSE: Thank you – This is well noted, and the Figure 1 and data analysis edits have been added as previously described.

4. Is the manuscript presented in an intelligible fashion and written in standard English?
Yes, overall I felt like the manuscript is very well written, presented clearly, and that the authors did a great job with their discussion section. I had two comments regarding this section

A) On page 6 line 86 of the manuscript draft the authors include the word "medial" as an example of a significant condition for exclusion criteria. Is this word intended to be "medical"?
RESPONSE: Thank you for noting this. The word "medial" was a typographical error and has been corrected to "medical" in the revised manuscript.

B) On page 9 line 150 of the manuscript the authors state that they used "Byers" method for incidence rate confidence interval calculations. Was this meant to be written as "Byar's" method, which I believe is the recognized standard spelling? This "Byer's" spelling is also used in Table 2 on page 13 of the draft manuscript.
RESPONSE: The reviewer was correct: Byar's method was used. The manuscript has been updated accordingly.

Reviewer #2:
Question 1: Yes

This is a well-designed prospective cohort study examining HIV incidence among adults with multiple sexual partners in a high-burden setting in Western Kenya. Overall, the study is technically sound and clearly presented. The data support the authors' primary conclusions, which are presented with humility and are not overreaching.

The longitudinal design, frequent HIV testing using what look like pre-established algorithms, and standardized behavioral data collection are appropriate. Laboratory procedures appear rigorous, and outcome ascertainment is well explained. Ethical oversight is strong (both Kenyan and U.S. IRBs). I appreciated the explanation of informed consent procedures.

The inclusion of a masking group of participants living with HIV is explained (HIV-related stigma in the community). However, this aspect of the design raises important questions. Perhaps a brief statement clarifying that these participants were aware of their HIV status, received appropriate counseling and linkage to care, and were aware that they were being enrolled as controls would help reassure readers less familiar with this approach.

RESPONSE: Thank you for highlighting this important aspect. We have added a brief statement in the Methods, as follows:
***Participants in the masking group were aware of their HIV status, provided informed consent, received appropriate counseling and linkage to care, and understood that they were being enrolled as controls for laboratory assays* (Lines 88-90).**

Question 2: Yes

The statistical analyses are appropriate for the study design. Incidence rates are calculated using standard person-time methods suitable for rare events, and the authors do a good job of explaining incorporation of pandemic considerations. Cox proportional hazards models are used appropriately, and violations of proportional hazards assumptions are clearly stated. I appreciated that the authors avoid overfitting and interpret adjusted analyses cautiously.

The low number of incident HIV infections is an important limitation and is acknowledged. That said, the observed incidence remains higher than national estimates cited in the Background. This raises the question of whether anticipated incidence informed enrollment targets. Power/sample size calculations are not presented, so inclusion of a brief statement describing enrollment rationale or incidence assumptions (if there were any) would help readers better contextualize the study's power to detect associations (or even whether this was considered in the study design).

Several points of clarity could strengthen the manuscript. In the Introduction, the discussion of "assessing the feasibility of future HIV vaccine and therapeutic trials" is somewhat unclear and does not clearly flow into the Results or Discussion. This should either be more explicitly addressed later in the manuscript or removed from the abstract and introduction. My first read of the manuscript was in the "assessing the feasibility" mindset, and I do not think this was an obvious throughline. A smaller point: for those of us less familiar, the description of fisherfolk would benefit from a brief explanation of their unique characteristics, especially if they represent a large portion of the study population.
RESPONSE: Thank you for the positive feedback. Regarding anticipated incidence and enrollment targets, the study's enrollment goal was informed by expected HIV incidence of approximately 3% per year, and the sample size was selected to ensure adequate precision for estimating incidence and retention in this high-risk population. This has been clarified in the text as follows: *An enrollment target of 600 participants without HIV was set based on a hypothesized HIV incidence of 3 cases per 100 person-years. Assuming retention of 85% of participants, 0 cases would need to be observed over 925 person-years in order to conclude with 95% confidence that the true HIV incidence was no less than hypothesized (95% confidence interval 3.1-5.9 cases per 100 person-years). Participants who were lost to follow-up while the study was still open for accrual could be replaced at investigator discretion.* (Lines 101-106).

Reference to "assessing the feasibility of future HIV vaccine and therapeutic trials" has been removed and the Introduction portion of the abstract now states: *We estimated HIV incidence among adults with multiple sexual partners and identified risk factors associated with HIV acquisition.*

The comment on fisherfolk was very helpful. The description of fisherfolk has been expanded slightly to highlight characteristics relevant to HIV risk. The additional text in the Introduction reads:
***Additionally, fisherfolk in this region have been shown to experience high mobility, transactional sexual networks, and elevated HIV prevalence, making them an important population in local HIV epidemiology* (Lines 61-63).**

In the Methods and Results, clearer wording would improve readability. For example, line 40: the variable describing unwanted sexual events should explicitly include the term "forced." It is used in the table, but should be used here as well. By not including the word "forced," this variable casts a much wider net. You should be clear here what you are implying, which I think is rape, assault, or coercion. Consistent formatting of numbers throughout the Results (particularly lines 171-174) would also improve readability. Consideration could also be given to including literacy in baseline characteristics if these data were collected, though education is used and is probably getting at the same thing.
RESPONSE: Thank you for these helpful suggestions. We have edited the manuscript where appropriate to ensure clearer wording. Specifically, the variable describing unwanted sexual events now explicitly uses the term "forced sexual activity" for consistency with Table 1. And we agree that educational attainment captures a closely related construct of literacy, thus literacy was not added as a separate characteristic.

In the Discussion, several interpretations could be sharpened. Comparisons with other incidence studies (lines 220-224) would benefit from consistent use of rates per 1000 person-years. Paragraph 219-228 raises the question of whether there have been similar studies in which incidence was markedly higher, or if low incidence is a persistent limitation in these types of studies. In lines 230-233, it may also be worth noting that individuals who self-select into longitudinal studies and are able to commit to follow-up may be more likely to adopt safer sexual behaviors over time. Additionally, the statement in lines 238-239 is too strong for something not statistically significant. I recommend dropping "This trend is consistent with" (line 239) and starting the sentence with "Regional…".
RESPONSE: Thank you for these valuable suggestions. We agree that consistent use of incidence rates per 1000 person-years would be ideal, therefore, we have updated all cited rates to indicate cases per 1000 person-years.
With respect to the observed low incidence, most similar cohorts have also reported lower-than-anticipated rates, often attributed to heightened prevention behaviors resulting from study participation and ongoing intensified HIV prevention interventions, including PEPFAR-supported programs in high-burden regions. An explanation has been added to the

Discussion as follows: ***Similar longitudinal cohorts in high-burden regions have also reported lower-than-anticipated HIV incidence, often attributed to a cohort effect of heightened prevention behaviors resulting from study participation as well as intensified HIV prevention efforts in the community, including PEPFAR-supported interventions (Line 241-244).***
Per your suggestion, we have also added the point that participants who self-select into longitudinal studies and maintain follow-up may adopt safer behaviors over time. The new text reads: ***Additionally, individuals who self-select into longitudinal studies and remain engaged in follow-up may adopt safer sexual behaviors over time, contributing to declining incidence in later study periods*** **(Line 263-265).**
Finally, we revised the phrasing by removing "This trend is consistent with…" and beginning the sentence with "Regional…" as recommended.

Question 3: No

The authors state that restrictions will apply. They do have appropriate statements for data availability and requests.

Question 4: Yes

Overall, this is a solid and carefully conducted study that adds useful incidence data from a high-burden setting. The results are well presented, and the discussion is humble and even-handed. The suggested revisions are largely clarifying in nature and would further strengthen the manuscript without altering its main conclusions.
RESPONSE: Thank you for the remarks and input. The manuscript has been updated with the considerations highlighted.

---

6. PLOS authors have the option to publish the peer review history of their article (what does this mean?). If published, this will include your full peer review and any attached files.

If you choose "no", your identity will remain anonymous but your review may still be made public.

Do you want your identity to be public for this peer review? For information about this choice, including consent withdrawal, please see our Privacy Policy.
Reviewer #1: No
Reviewer #2: No

[NOTE: If reviewer comments were submitted as an attachment file, they will be attached to this email and accessible via the submission site. Please log into your account, locate the manuscript

record, and check for the action link "View Attachments". If this link does not appear, there are no attachment files.]

To ensure your figures meet our technical requirements, please review our figure guidelines: https://journals.plos.org/plosone/s/figures
You may also use PLOS's free figure tool, NAAS, to help you prepare publication quality figures: https://journals.plos.org/plosone/s/figures#loc-tools-for-figure-preparation.
NAAS will assess whether your figures meet our technical requirements by comparing each figure against our figure specifications.
